# Water Pipes

**Published:** 1879-05

**Authors:** 


### WATER PIPES.

It is becoming more common every year to supply farm- houses with water through lead pipes, distributed through the building. Some waters, as the Schuylkill, which supplies Phila- delphia, contain an element which forms on the inside of the pipe, a film which is absolutely impervious by the water, and pro- tects the lead against all corrosion or chemical change. And in cities and large towns, where the water is kept running almost incessantly, time is not allowed for chemical action on the lead; where the same water, through the same pipes, would produce speedy sickness in a farm-house. It is water stagnant in a lead pipe which causes mischief; so that every faucet should be allowed to run the water waste for at least one minute the first thing in the morning, especially in the kitchen.

